# 21968 Adapting a global telehealth model to solve U.S. healthcare needs: age-related hearing loss as a test market

**DOI:** 10.1017/cts.2021.509

**Published:** 2021-03-30

**Authors:** Samuel Weinreb, Izabella Samuel, Kai Zhang, Mackenzie Hall, Mariana Bendavit, Adler Archer, Neha Verma

**Affiliations:** 1 Johns Hopkins University School of Medicine; 2 Johns Hopkins Center for Bioengineering Innovation and Design

## Abstract

ABSTRACT IMPACT: We are adapting a global telehealth platform and model of care to the U.S. context in order to solve the problem of undertreatment of age-related hearing loss and, in turn, facilitate healthy aging and social engagement among older adults. OBJECTIVES/GOALS: Intelehealth is a nonprofit startup that provides medical care to last-mile populations in India by equipping frontline health workers with an open-source digital assistant and telemedicine platform. Here, we explore how this technology and model of care might be adapted to address health inequities in the context of the U.S. healthcare system. METHODS/STUDY POPULATION: We first sought to identify a specific healthcare need that we could address as a case study on applying the Intelehealth model more broadly in the U.S. context. We began with a needs assessment, wherein we conducted primary ethnographic research, expert interviews, and literature review to identify problems in the general areas of health disparities, community health workers, and telemedicine accessibility. We then scored each need on clinical impact, feasibility, business potential, and strategic fit. After a top need was selected, a root cause analysis was performed. Brainstorming and solution concepting will be followed by prototyping, iterative design with primary stakeholder feedback, usability testing, and finally implementation and validation of the solution. RESULTS/ANTICIPATED RESULTS: Of 106 needs, the most highly scored was undertreatment of age-related hearing loss (ARHL). The third most common chronic condition in the U.S., ARHL presents a significant barrier to healthy aging and the single largest modifiable risk factor for dementia; yet only 15% of those with ARHL regularly use hearing aids. Thus, a large market segment - nearly 30 million Americans - is underserved by the current hearing care paradigm. Root cause analysis revealed that the primary reasons for hearing aid non-use include stigma around aging, denial of hearing loss, poor awareness of resources, and insufficient education around proper use and maintenance. These barriers, being primarily sociocultural in nature, may be optimally addressed by community health workers, making ARHL an ideal fit for the Intelehealth model. DISCUSSION/SIGNIFICANCE OF FINDINGS: We have identified ARHL as an optimal test market for Intelehealth in the U.S. By developing a targeted intervention to improve hearing aid access and acceptability among older adults, we will create a generalizable model for delivering care through community health workers equipped with a decision support and telemedicine platform.

